# Characterizing Extracellular Vesicles and Particles Derived from Skeletal Muscle Myoblasts and Myotubes and the Effect of Acute Contractile Activity

**DOI:** 10.3390/membranes12050464

**Published:** 2022-04-26

**Authors:** Benjamin Bydak, Taiana M. Pierdoná, Samira Seif, Karim Sidhom, Patience O. Obi, Hagar I. Labouta, Joseph W. Gordon, Ayesha Saleem

**Affiliations:** 1 Applied Health Sciences, University of Manitoba, Winnipeg, MB R3T 2N2, Canada; umbydakr@myumanitoba.ca (B.B.); obip@myumanitoba.ca (P.O.O.); 2 Children’s Hospital Research Institute of Manitoba (CHRIM), Winnipeg, MB R3T 2N2, Canada; taianapierdona@gmail.com (T.M.P.); samira.seif@umanitoba.ca (S.S.); hagar.labouta@umanitoba.ca (H.I.L.); joseph.gordon@umanitoba.ca (J.W.G.); 3 Faculty of Kinesiology and Recreation Management, University of Manitoba, Winnipeg, MB R3T 2N2, Canada; 4 Rady Faculty of Health Sciences, College of Medicine, University of Manitoba, Winnipeg, MB R3T 2N2, Canada; sidhomk@myumanitoba.ca; 5 Rady Faculty of Health Sciences, College of Pharmacy, University of Manitoba, Winnipeg, MB R3T 2N2, Canada; 6 Diabetes Research Envisioned and Accomplished in Manitoba (DREAM) Theme, Winnipeg, MB R3T 2N2, Canada; 7 Biology of Breathing (BoB) Theme, Winnipeg, MB R3T 2N2, Canada; 8 Rady Faculty of Health Sciences, College of Nursing, University of Manitoba, Winnipeg, MB R3T 2N2, Canada

**Keywords:** extracellular vesicles, extracellular particles, skeletal muscle, myoblasts, myotubes, differentiation, acute contractile activity, secretome, differential ultracentrifugation, total exosome isolation kit

## Abstract

Extracellular vesicles (EVs), released from all cells, are essential to cellular communication and contain biomolecular cargo that can affect recipient cell function. Studies on the effects of contractile activity (exercise) on EVs usually rely on plasma/serum-based assessments, which contain EVs from many different cells. To specifically characterize skeletal muscle–derived vesicles and the effect of acute contractile activity, we used an in vitro model where C2C12 mouse myoblasts were differentiated to form myotubes. EVs were isolated from conditioned media from muscle cells at pre-differentiation (myoblasts) and post-differentiation (myotubes) and also from acutely stimulated myotubes (1 h @ 14 V, C-Pace EM, IonOptix, Westwood, MA, USA) using total exosome isolation reagent (TEI, ThermoFisher (Waltham, MA, USA), referred to as extracellular particles [EPs]) and differential ultracentrifugation (dUC; EVs). Myotube-EPs (~98 nm) were 41% smaller than myoblast-EPs (~167 nm, *p* < 0.001, *n* = 8–10). Two-way ANOVA showed a significant main effect for the size distribution of myotube vs. myoblast-EPs (*p* < 0.01, *n* = 10–13). In comparison, myoblast-EPs displayed a bimodal size distribution profile with peaks at <200 nm and 400–600, whereas myotube-Eps were largely 50–300 nm in size. Total protein yield from myotube-EPs was nearly 15-fold higher than from the myoblast-EPs, (*p* < 0.001 *n* = 6–9). Similar biophysical characteristics were observed when EVs were isolated using dUC: myotube-EVs (~195 nm) remained 41% smaller in average size than myoblast-EVs (~330 nm, *p* = 0.07, *n* = 4–6) and had comparable size distribution profiles to EPs isolated via TEI. Myotube-EVs also had 4.7-fold higher protein yield vs. myoblast EVs (*p* < 0.05, *n* = 4–6). Myotube-EPs exhibited significantly decreased expression of exosomal marker proteins TSG101, CD63, ALIX and CD81 compared with myoblast-EPs (*p* < 0.05, *n* = 7–12). Conversely, microvesicle marker ARF6 and lipoprotein marker APO-A1 were only found in the myotube-EPs (*p* < 0.05, *n* = 4–12). There was no effect of acute stimulation on myotube-EP biophysical characteristics (*n* = 7) or on the expression of TSG101, ARF6 or CD81 (*n* = 5–6). Myoblasts treated with control or acute stimulation–derived EPs (13 µg/well) for 48 h and 72 h showed no changes in mitochondrial mass (MitoTracker Red, ThermoFisher, Waltham, MA, USA), cell viability or cell count (*n* = 3–4). Myoblasts treated with EP-depleted media (72 h) exhibited ~90% lower cell counts (*p* < 0.01, *n* = 3). Our data show that EVs differed in size, distribution, protein yield and expression of subtype markers pre vs. post skeletal muscle–differentiation into myotubes. There was no effect of acute stimulation on biophysical profile or protein markers in EPs. Acute stimulation–derived EPs did not alter mitochondrial mass or cell count/viability. Further investigation into the effects of chronic contractile activity on the biophysical characteristics and cargo of skeletal muscle–specific EVs are warranted.

## 1. Introduction

Extracellular vesicles (EVs) were initially discovered in two back-to-back 1983 papers on the recycling of the transferrin receptor in small vesicles released from rat and sheep reticulocytes [1,2]. The term “exosomes”, coined by Dr. Rose Johnstone a few years later, refers to what is now known as the smallest family member of EVs [3]. An evolutionarily conserved mode of communication, EVs are secreted by all types of cells [4] and are found in all biological fluids [5], such as blood [6,7], saliva [8], urine [9], breast milk [10], human semen [11] and cerebrospinal fluids [12]. EVs enclose distinct biological cargo that can be modified depending on alterations in the cellular milieu [13] and can in turn modulate recipient cell function [14]. Classically, EVs are broadly divided into three separate groups [15,16,17,18]: exosomes (30–150 nm), microvesicles (100–1000 nm) and apoptotic bodies (1000–5000 nm) and can be isolated using various techniques, as we have previously reviewed [19]. Current MISEV guidelines recommend classification of EVs according to (1) size as small EVs (<200 nm) or medium/large EVs (>200 nm), (2) density, (3) biochemical marker composition or (4) by cell of origin/environmental milieu [19]. Irrespective of how they are named, strong evidence shows that EVs mediate the crosstalk between organs and tissues to facilitate the coordination and propagation of physiological changes [16].

In 2006–2007, miRNA was identified within EVs [20,21] and research interest in EVs skyrocketed once it was established that EVs could transfer nucleic acids between cells. It was not until the late 2000 s that studies began to find that EVs could potentiate intercellular communication via transportation of cargo, including RNA, protein, membrane receptors and others [17,22,23,24,25]. Since then, EVs have been extensively studied as biomarkers of chronic and acute diseases [26,27,28,29,30,31,32,33,34], as therapeutic vectors for drug delivery [35,36,37] and more recently in physiological contexts.

Skeletal muscle accounts for ~40% of body mass, and serves as an endocrine organ able to secrete a multitude of proteins, lipids and metabolites (i.e., myokines) that are released from muscle upon physical activity, and it is essential in mediating some of the systemic effects of exercise [22]. While a plethora of studies have illustrated the endocrine function of skeletal muscle in vivo [38], the first in vitro report to show unequivocal evidence for contraction-induced myokine secretion from skeletal muscle cells in culture was only recently published [39]. Myokines can be released through the classical signaling pathway, and also secreted packaged within EVs. In fact, nearly 400 proteins have been identified in EVs from skeletal muscle alone, and many well-established myokines were found inside EVs [16,40,41,42,43]. Several papers demonstrated that skeletal muscle cells are capable of releasing EVs in culture, both from undifferentiated myoblasts and differentiated myotubes [40,41,44] and that myotube-derived EVs can modulate recipient cell function [42,44]. Given the central role of skeletal muscle in exercise-induced adaptations and whole-body regulation of metabolism, determining the role of exercise-evoked skeletal muscle–derived EVs (Skm-EVs) is critical. While many studies have shown an increase in systemic EVs during exercise in particular [45,46,47,48,49], identifying the contribution of Skm-EVs is challenging for several reasons: (1) markers used for skeletal muscle, e.g., alpha-sarcoglycan [50], while abundant in muscle, cannot be guaranteed to be expressed in all Skm-EVs, (2) the difficulty in confirming that the Skm-EVs originated from the muscle undergoing contractile activity and (3) intramuscular injections of fluorescent-labeled EVs or the use of genetic manipulation, while excellent options in rodent studies, are not viable approaches for human exercise studies. This underscores the importance of using in vitro models of Skm-EVs where contractile activity can be used to mimic exercise in order to comprehensively characterize Skm-EVs and determine their role in juxtracrine, autocrine and endocrine signaling.

Here, we compared EV characterization as a function of skeletal muscle myotube formation with acute contractile activity. Murine (C2C12) skeletal muscle myoblasts were differentiated into myotubes and then electrically stimulated using IonOptix C-PACE EM. Conditioned media were collected pre- and post-myotube formation to isolate vesicles using total exosome isolation reagent (TEI, ThermoFisher) and differential ultracentrifugation (dUC). We refer to particles isolated via TEI as extracellular particles (EPs) and those via dUC as EVs as a nod to the lack of specificity of the former when compared to the latter, as recommended in the MISEV guidelines [51]. EVs and EPs were isolated and characterized for biophysical properties and expression of marker proteins in accordance with MISEV guidelines [51]. Next, we electrically paced myotubes and isolated EPs from the conditioned media. Electrical stimulation of cultured myotubes is an established method of evoking contractile activity in vitro [52]. However, it does not capture the complexity of acute exercise nor can it fully recapitulate the heterogeneity of the tissues involved, the fiber types, or the size principle of fiber type recruitment upon contraction. Nevertheless, this model can potentiate the effects observed with traditional exercise training, i.e., an increase in mitochondrial biogenesis in C2C12 myotubes [53,54,55]. Accordingly, it has been used as a surrogate for both acute and chronic exercise in vitro, where the goal is to evaluate the function of skeletal muscle in contraction-induced mitochondrial biogenesis, the hallmark adaptation to exercise training. Hence, we used this model to specifically measure the effects of contractile activity on skeletal muscle–derived vesicles and assessed the biological activity of the vesicles derived post-stimulation to those derived from unstimulated control cells. To do this, we co-cultured EPs isolated post-acute stimulation with myoblasts and measured changes in mitochondrial content and cell viability.

## 2. Materials and Methods

### 2.1. C2C12 Myoblast Proliferation and Differentiation into Myotubes

C2C12 myoblasts were seeded at 90,000 cells/well in a 6-well dish and grown in fresh DMEM (Corning^®^, Corning, NY, USA) supplemented with 10% fetal bovine serum (FBS) (ThermoFisher, Waltham, MA, USA) and 1% penicillin/streptomycin (P/S) (Corning^®^, Corning, NY, USA), as previously described [54,56]. After 24 h, conditioned media from myoblasts were collected and used for vesicle isolation. Upon reaching 95% confluency, myoblasts were placed in differentiation media (DMEM, 5% horse serum, 1% P/S) for 5 days to obtain fully differentiated myotubes. Conditioned media from myotubes were collected on day 6 and used for vesicle isolation. Myotubes were electrically stimulated on day 7 as described below to induce acute contractile activity.

### 2.2. Skeletal Muscle EP Isolation

Conditioned media were immediately centrifuged at 2000× *g* to remove cell debris. The pellet was discarded, and supernatant was used for EP isolation using TEI kit (ThermoFisher, cat #4478359, Waltham, MA, USA) according to the manufacturer’s instructions and as described before [44]. Modifications to the protocol included nutating conditioned media with 0.5 volume of TEI solution overnight at 4 °C (16 h). After nutation, samples were centrifuged at 10,000× *g* for 1 h (4 °C), washed with 4 mL PBS (Corning^®^, Corning, NY, USA) and centrifuged again at 10,000× *g* for 1 h at 4 °C. Pellets were re-suspended in 70 µL PBS. The supernatant was used for EP-depleted media treatment. Protein concentration of isolated EPs was determined using commercially available BCA protein assay kit (Pierce™, ThermoFisher, Waltham, MA, USA) as previously described [57]. Protein yield was calculated by multiplying the concentration by the total volume of EP isolates.

### 2.3. Skeletal Muscle–EV Isolation

We used dUC to isolate EVs following the protocol by Théry et al. [58]. Media were spun at 300× *g* for 10 min at 4 °C to pellet dead cells, followed by 2000× *g* for 10 min at 4 °C and then 10,000× *g* for 30 min at 4 °C to remove cellular debris and large vesicles, respectively. The supernatant was then centrifuged at 100,000× *g* for 70 min at 4 °C using Sorvall™ MTX 150 Micro-Ultracentrifuge, S58-A fixed angle rotor (ThermoFisher, Waltham, MA, USA) to obtain the exosome/small EV pellet. EV pellet was resuspended in 1 mL PBS and centrifuged again at 100,000× *g* for 70 min at 4 °C. The final pellet was resuspended in 50 μL PBS and used for subsequent analysis. Protein concentration of isolated EVs was determined using commercially available BCA protein assay kit (Pierce™, ThermoFisher, Waltham, MA, USA) as previously described [57]. Protein yield was calculated by multiplying the concentration with total volume of EV isolates.

### 2.4. Size and Zeta Analysis

The hydrodynamic diameter (size) and zeta potential of EPs and EVs was characterized by phase analysis light scattering (NanoBrook ZetaPALS, Brookhaven Instruments, Holtsville, NY, USA) instrument in collaboration with Dr. Hagar Labouta’s lab (College of Pharmacy, University of Manitoba). Access to core facility housing ZetaPALS (NanoBrook, Brookhaven Instruments, Holtsville, NY, USA) was limited at times and thus necessitated isolated vesicles to be stored for up to 24–48 h at 4 °C before characterization. EPs/EVs were diluted 1:75 in PBS and kept on ice until analysis. Each sample underwent 5 runs, with each run ~15 s, with a dust cut-off set at 40. Size was measured as an intensity averaged multimodal distribution using a scattering angle of 90°, and size bins were used to represent total size intensity within a given size range. Zeta potential analysis was performed using a Solvent-Resistant Electrode (NanoBrook, Brookhaven Instruments, Holtsville, NY, USA) and BI-SCGO 4.5 mL cuvettes (NanoBrook, Brookhaven Instruments, Holtsville, NY, USA). For zeta potential, each sample was loaded into the cuvette, and the electrode inserted for phase analysis light scattering to carry out mobility measurements. Values were averaged (irrespective of negative/positive charge) to calculate zeta potential using the Smoluchowski formula from mobility measurements [59]. All measurements were performed in PBS (pH 7.4) at 25 °C.

### 2.5. Western Blotting

For immunoblotting, 20 μg of myoblast/myotube-EPs, and 50 μg of stimulated (STIM) and non-stimulated control myotube-EP lysate were denatured with 5% solution of β-mercaptoethanol, incubated at 95 °C for 5 min, then loaded on Mini-PROTEAN® TGX™ Precast Gels (Bio-Rad, Hercules, CA, USA) for 15 min at 300 V. Due to the extremely low levels of TSG101 observed in myotube-EPs, we decided to run Western blots with higher total protein content per lane (50 µg) for subsequent experiments. Proteins were transferred to polyvinylidene difluoride membrane using a Trans-Blot^®^ Turbo™ (Bio-Rad, Hercules, CA, USA). Once transferred, membranes were washed with TBS-Tween20 (TBST) for 10 min and blocked with 5% skim milk in TBST solution for 2 h at room temperature. Membranes were incubated with the primary antibodies against target proteins: rabbit polyclonal anti-TSG 101 (T5701, Sigma-Aldrich Co., Saint Louis, MO, USA, 1:200), rabbit polyclonal anti-CD63 (SAB4301607, Sigma-Aldrich Co., Saint Louis, MO, USA, 1:1000), rabbit monoclonal anti-Alix (MCA2493, Bio-Rad, Hercules, CA, USA, 1:500), mouse monoclonal anti-CD81 (sc-166029, Santa Cruz Biotechnology, Dallas, TX, USA, 1:200), mouse monoclonal anti-ARF6 (sc-7971, Santa Cruz Biotechnology, Dallas, TX, USA, 1:200), mouse monoclonal anti-Apolipoprotein A1 (0650-0050, Bio-Rad, Hercules, CA, USA 1:200), rabbit polyclonal anti-Cytochrome C (AHP2302, Bio-Rad, Hercules, CA, USA 1:200) and mouse monoclonal anti-β-actin (A5441, Sigma-Aldrich Co., Saint Louis, MO, USA 1:5000) in 1% skim milk overnight at 4 °C. Membranes were washed 3x with TBST and incubated with anti-mouse or anti-rabbit IgG horseradish peroxidase secondary antibody (A16017 or A16035, ThermoFisher, Waltham, MA, USA) at dilution of 1:1000–10,000 for 1 h. Membranes were visualized by enhanced chemiluminescence detection reagent (Bio-Rad, Hercules, CA, USA) and imaged using a ChemiDoc System (Bio-Rad, Hercules, CA, USA). Some membranes were stripped and re-probed for analysis of target proteins. To accomplish this, membranes were washed 3x with TBST, placed in petri dishes with Restore™ stripping buffer (ThermoFisher, Waltham, MA, USA) for 30 min at room temperature, washed with TBST to remove stripping buffer and incubated with primary and secondary antibodies against protein targets as described above. Band densities of all measured proteins were normalized to the respective Coomassie staining of gels as a loading control. 

### 2.6. Acute Stimulation (STIM) of C2C12 Myotubes

Electrical stimulation of myotubes has been used to evoke contractile activity and mimic exercise in vitro, as shown previously [53,54,56]. Prior to STIM, IonOptix C-Dish, stim electrode plates (IonOptix LLC, Westwood, MA, USA), were sterilized in UV light for 30 min. After sterilization, two electrode plates were loaded into two 6-well dishes with fully differentiated myotubes ready for contractile activity. Once loaded, a single plate (designated as STIM) was connected to the stim machine, IonOptix C-Pace EM (IonOptix LLC, Westwood, MA, USA), whereas the control plate (designated as non-stimulated controls) was not connected to the IonOptix C-Pace EM. Stimulation was performed at 14 V and 1 Hz for 1 h, while both plates were incubated at 37 °C. Immediately after STIM, media were collected, and EP isolation was performed as described above using TEI. 

### 2.7. Treatment of C2C12 Myoblasts with EPs Isolated from Control and STIM Myotubes

90,000 myoblasts/well were seeded in 6-well plates in 2 mL/well of growth media. 6.67 µg/mL EPs (total 13 µg EPs/well) or 1 mL EP-depleted media from control or STIM myotubes was added to myoblasts when cells were at 80–90% confluency. Cells were treated for 48 h and 72 h at 37 °C. After treatment, media were discarded and treated myoblasts collected for MitoTracker Red CMXRos staining (ThermoFisher, Waltham, MA, USA), cell count or viability assays, as described below.

### 2.8. MitoTracker Staining

MitoTracker Red CMXRos (Cell Signaling, Danvers, MA, USA, #9082) was first prepared to a concentration of 0.1 M in 1× PBS. 60 µL of 0.1 M of MitoTracker Red CMXRos was then mixed in 12 mL of growth media to prepare staining solution. Cells were then washed twice with 1× PBS, stained with 800 µL of staining solution and incubated at 37 °C for 30 min. Following incubation, cells were washed 2× with PBS and covered with 1 mL of growth media before imaging using epifluorescence microscopy (Zeiss Axiovert 200, White Plains, NY, USA).

### 2.9. Cell-Count and Viability Assay

Cells were washed twice with 800 µL PBS, trypsinized with 400 µL of trypsin and incubated for 3 min at 37 °C. 1 mL of growth media was added, and cells were centrifuged at 1000× g for 5 min to pellet the cells. Pelleted cells were stained with a 1:1 dilution of 0.4% Trypan blue solution (Sigma-Aldrich Co., cat #T8154, Saint Louis, MO, USA), then counted with a hemocytometer (Hausser Scientific Bright-Line Hemacytometer, Sigma-Aldrich Co., Saint Louis, MO, USA). Total number of cells was counted and expressed per mL of growth media. Cell viability was obtained by dividing the number of live cells (not stained by the trypan) by the total number of counted cells.

### 2.10. Statistical Analysis

All data were analyzed using an unpaired Student’s *t* test. Size distribution and cell count were assessed using a two-way ANOVA with Bonferroni post hoc correction for multiple comparisons. Significance was set at *p* < 0.05. All data are presented as mean ± standard error. Statistical analysis was performed using GraphPad Prism software (version 8.4.2, GraphPad, San Diego, CA, USA).

## 3. Results

### 3.1. Isolated Vesicles Differed in Size, Protein Yield and Expression of Vesicle Subtype Markers When Isolated Pre- and Post-Differentiation into Myotubes

**Figure 1:**
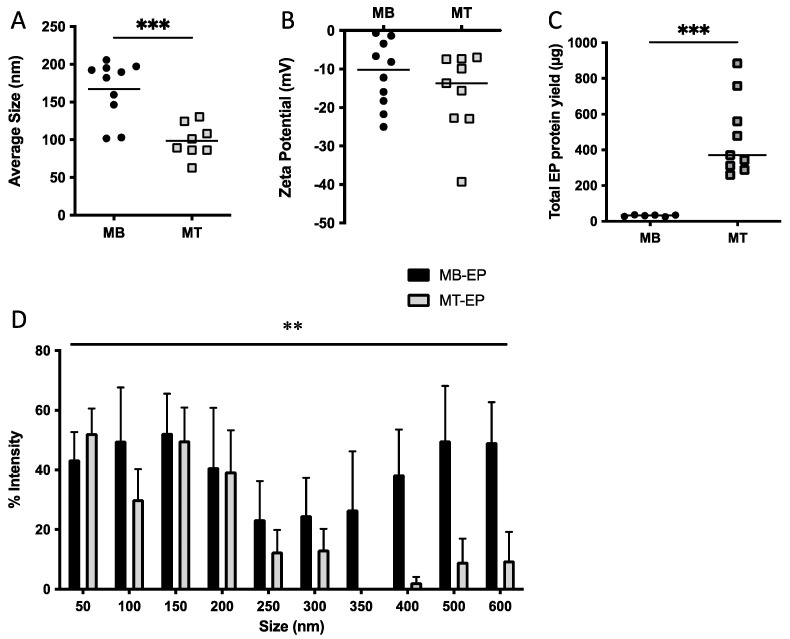
Changes in EP biophysical characteristics with skeletal muscle myoblast differentiation into myotubes. (**A**) C2C12 myotube (MT) EPs were 41% smaller than myoblast (MB) EPs (*** *p* < 0.001, *n* = 8–10). (**B**) Zeta potential remained unchanged between EPs from myoblasts vs. myotubes, *n* = 9–10. (**C**) Total protein yield from myotube-EPs was ~15-fold higher than myoblast-EPs (*** *p* < 0.001, *n* = 6–9). (**D**) Two-way ANOVA showed a significant main effect for myotube-EPs vs. myoblast-EPs (** *p* < 0.01, *n* = 10–13). Myoblast-EPs display a bimodal size distribution profile with a peak for EPs < 200 nm and again at 400–600 nm, compared to myotube-EPs that were enriched with 50–300 nm sized particles. EPs were isolated using Total Exosome Isolation Reagent (ThermoFisher, Waltham, MA, USA). All data were analyzed using an unpaired Student’s *t* test, except in Figure 1D where a 2-way ANOVA was used. Data are expressed as scatter plots showing mean, or bars with mean ± standard error.

**Figure 2:**
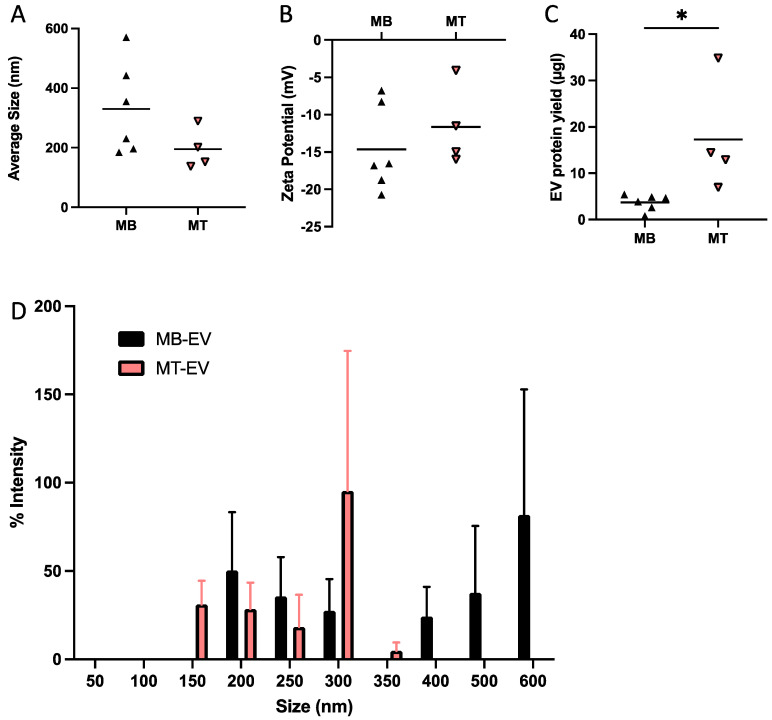
Differences between myoblast (MB) vs. myotube (MT) EV biophysical characteristics when isolated using differential ultracentrifugation (dUC). C2C12 myoblast- and myotube-EVs isolated via dUC showed no difference in (**A**) average EV size or (**B**) zeta potential (*n* = 4–6). (**C**) Myotube EV protein yield was 4.79-fold higher than myoblast EV protein yield (* *p* < 0.05, *n* = 4–6). (**D**) Myoblast EVs display a bimodal size distribution profile with an increase at 200 nm and again at 600 nm, compared to myotubes EVs that were largely enriched with EVs 150–300 nm (*n* = 4–6). All data were analyzed using an unpaired Student’s *t* test, except in Figure 2D where a 2-way ANOVA was used. Data are expressed as scatter plots showing mean, or as bar graphs with mean ± standard error.

Average myotube-EP size (98 nm) was 41% smaller than myoblast-EPs (167 nm, *p* < 0.001, Figure 1A). The smallest particle size was 63 nm for myotube-EPs vs. 102 nm for myoblast-EPs, while the maximum size observed was 130 nm for myotube-EPs vs. 206 nm for myoblast-EPs. There was no difference in zeta potential between the two groups (Figure 1B). Total protein yield from myotube-EPs was ~15-fold higher than myoblast-EPs (*p* < 0.001, Figure 1C). A two-way ANOVA on size distribution between myoblast-EPs and myotube-EPs showed a significant main effect for cell type (*p* < 0.05, Figure 1D). Myoblast-EPs (represented as black bars) show a bimodal EV size distribution pattern with increase in expression at <200 nm and at 400–600 nm (Figure 1D). In contrast, myotube-EPs (represented as gray bars) are largely enriched with 50–300 nm sized particles (Figure 1D). To compare our results with the gold standard method of EV isolation, we next used dUC to isolate EVs. Average EV size, distribution profile, and zeta potential were not statistically different between myotube-EVs and myoblast-EVs (Figure 2A,B,D), but protein yield was (Figure 2C). Despite this lack of statistical significance, we observed the same trends. Myotube-EVs (~195 nm) remained 41% smaller in average size than myoblast-EVs (~330 nm, *p* = 0.07, *n* = 4–6, Figure 2A). The minimum EV size was 138 nm for myotube-EVs and 184 nm for myoblast-EVs. The maximum EV size was 290 nm for myotube-EVs vs. 570 nm for myoblast-EVs. There was no difference in zeta potential between myoblast-EVs and myotube-EVs (Figure 2B). Myotube-EV protein yield was 4.79-fold higher than myoblast-EV protein yield (*p* < 0.05, Figure 2C), albeit overall protein yield was 20–40× lower in EVs isolated via dUC (Figure 2C) when compared to the TEI method of EP isolation (Figure 1C). Further, myoblast-EVs (represented as black bars) displayed a biomodal size distribution with enrichment of <200 nm and 400–600 nm sized vesicles (Figure 2D). Myotube-EVs (represented as pink bars) conversely were enriched with 150–300 nm sized EVs (Figure 2D). The high degree of variability in size distribution (Figure 2D) is likely due to the small sample size and the use of DLS to characterize EVs. Given that the patterns in EV characterization were largely similar in dUC vs. TEI-based methods, the flexibility of usage of TEI over dUC, and the fact that many laboratories do not have access to highly specialized infrastructure such as an ultracentrifuge or the investment capacity to purchase EV-depleted serum from commercial sources in perpetuity, we performed the remaining experiments using TEI-derived EPs.

**Figure 3:**
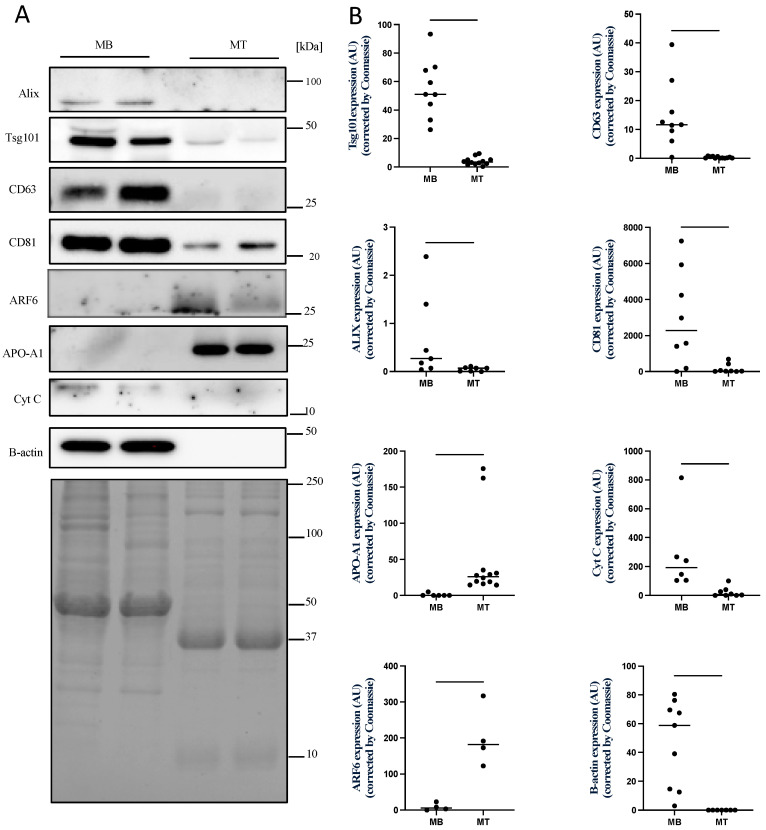
Effect of skeletal muscle myoblast differentiation into myotubes on the expression of proteins related to EV subtypes. (**A**) Representative immunoblots showing equal amounts (20 µg) of myoblast (MB) or myotube (MT) EP protein lysates were subjected to 12% SDS–PAGE. EPs were isolated using Total Exosome Isolation Reagent (ThermoFisher, Waltham, MA, USA). Coomassie blue gel staining was used as a loading control. (**B**) Quantification of immunoblot analysis showing the expression of different markers of small and medium/large EVs and non-EV co-isolates. Expression of small EV marker proteins TSG101, ALIX and tetraspanins CD63 and CD81 were enriched by several orders of magnitude in myoblast-EP preparations compared with myotube-EPs (* *p* < 0.05, ** *p* < 0.01, *** *p* < 0.001, **** *p* < 0.0001, *n* = 7–12). Conversely, levels of ARF6, a microvesicle marker, and APO-A1, a lipoprotein non-EV co-isolate, were only expressed in myotube-EPs when compared with myoblast-EPs (* *p* < 0.05, ** *p* < 0.01, *n* = 4–12). Cytochrome c and beta-actin were barely expressed in myotube-EPs compared to myoblast-EPs (** *p* < 0.01, *n* = 6–9). Data were analyzed using an unpaired Student’s *t* test and are expressed as scatter plots showing the mean.

Next, we compared the expression of protein markers commonly associated with small EVs or exosomes, i.e., TSG101, Alix, CD81 and CD63, and medium/large EV (i.e., microvesicle) marker ARF6 to characterize EPs in compliance with MISEV guidelines [51]. Myotube-EPs exhibited significantly decreased expression of small EV protein markers (TSG101, CD63, ALIX and CD81), often by several orders of magnitude compared to myoblast-EPs (*p* < 0.05, Figure 3A,B). Conversely, APO-A1 (lipoprotein) expression and ARF6 expression were highly enriched in myotube-EPs vs. myoblast-EPs (*p* < 0.05, Figure 3A,B). Lastly, cytochrome c (a mitochondrial marker used to identify medium/large EVs) and beta-actin (housekeeping control) were barely expressed at quantifiable levels in myotube-EPs compared to myoblast-EPs (*p* < 0.05, Figure 3A,B).

### 3.2. Acute Stimulation Does Not Affect Vesicle Size, Zeta Potential, Protein Yield or Expression of Vesicle Subtype Protein Markers

**Figure 4:**
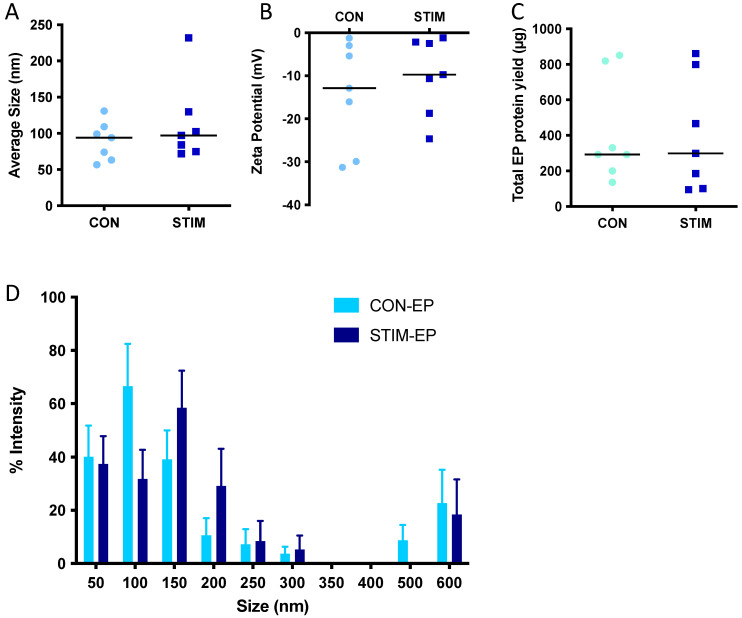
Effect of acute stimulation on EP size, zeta potential and protein yield. C2C12 myotubes were electrically contracted using an IonOptix EM-PACE for 1 h at 14 volts and EPs isolated from conditioned media. (**A**) Average size, (**B**) zeta potential and (**C**) protein yield remained unchanged between non-stimulated control (CON) and acutely stimulated (STIM) myotube-EPs (*n* = 7). (**D**) No statistically significant differences in size distribution was found between CON-EPs and STIM-EPs (*n* = 7). EPs were isolated using Total Exosome Isolation Reagent (ThermoFisher, Waltham, MA, USA). Data were analyzed using an unpaired Student’s *t* test, except for panel (**D**), which was analyzed by 2-way ANOVA. Data are expressed as scatter plots with means or bar graphs with mean ± standard error.

**Figure 5:**
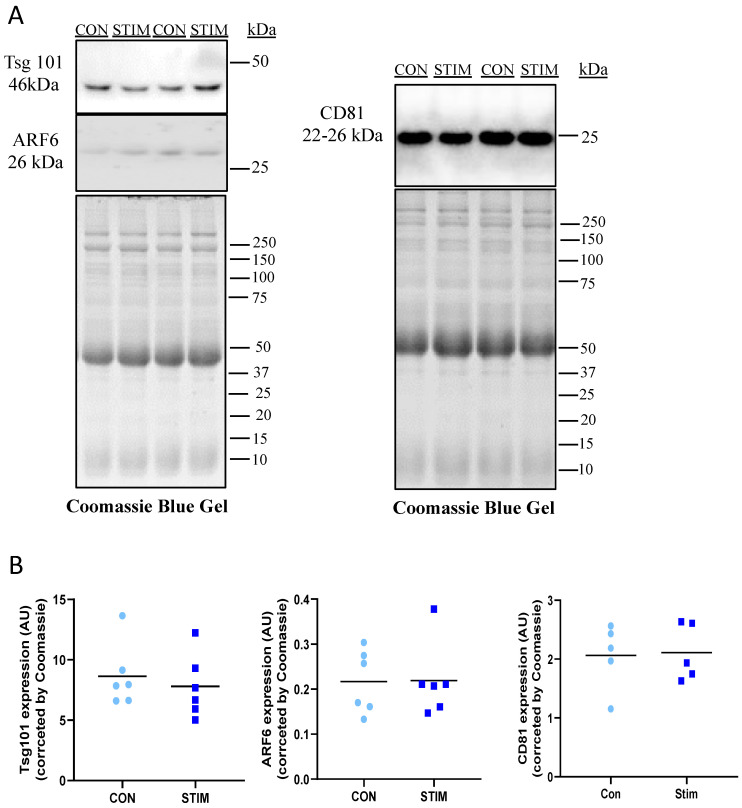
Effect of acute stimulation on expression of EV subtype protein markers. Equal amounts (50 µg) of non-stimulated control (CON) vs. acutely stimulated (STIM) myotube-EP lysates were subjected to 12% SDS–PAGE. EPs were isolated using Total Exosome Isolation Reagent (ThermoFisher, Waltham, MA, USA). Coomassie blue-stained gels were used as a loading control. (**A**) Representative immunoblots and (**B**) quantification of data shows no difference in the expression of exosomal markers CD81 and TSG101, nor in the content of microvesicle marker ARF6 in EPs lysates CON vs. STIM myotubes (*n* = 5–6). Data were analyzed using an unpaired Student’s *t* test and are expressed as scatter plots with mean.

To quantify the effects of acute contractile activity on myotube-EPs, fully differentiated myotubes were electrically paced at 14 V (1 Hz) for 1 h. Control myotubes were plated with the stimulatory electrode plate but did not receive stimulation. Immediately after stimulation, we collected conditioned media and performed EP isolation, as described earlier, using TEI. We found no statistically significant difference in average size (Figure 4A), zeta potential (Figure 4B), total protein yield (Figure 4C) or size distribution in control vs. stimulated conditions (Figure 4D). We evaluated the expression of small EV markers with stimulation. Due to the extremely low levels of TSG101 observed in myotube-EPs (Figure 3), we decided to run Western blots with higher total protein content per lane (50 µg) for these experiments (Figure 5). We found no difference in the expression of ARF6, TSG101 or CD81 in control vs. stimulated conditions (Figure 5A,B).

### 3.3. Effect of Vesicles Collected Post-Stimulation on Mitochondrial Content, Cell Count and Cell Viability

**Figure 6:**
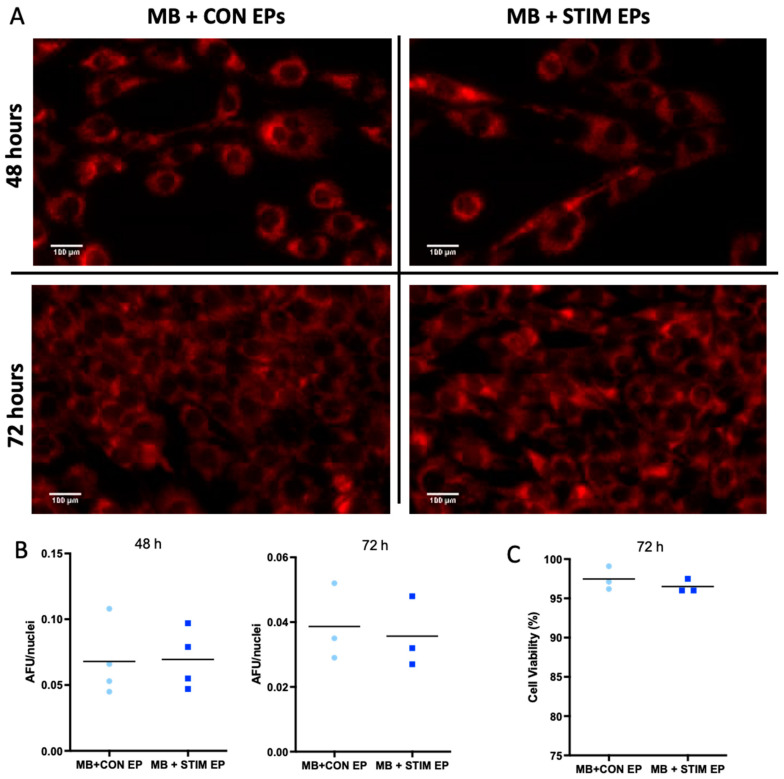
Mitochondrial content and cell viability in myoblasts (MB) treated with EPs from CON vs. STIM myotubes. Myoblasts were treated with EPs (13 µg/well) isolated from conditioned media (using Total Exosome Isolation Reagent (ThermoFisher, Waltham, MA, USA) from non-stimulated control (CON) vs. acutely stimulated (STIM) myotubes for 48 h and 72 h and changes in mitochondrial mass measured. (**A**) Representative fluorescent images of myoblasts stained with MitoTracker Red CMXRos after 48 h and 72 h of EP treatment from CON and STIM myotubes taken at 10× mag; scale bar = 100 µm. (**B**) Quantification of fluorescent images showed no change in mitochondrial content with STIM myotube-EP treatment. Images were normalized to nuclei count by dividing total fluorescence of each image by number of nuclei and expressed as arbitrary fluorescent units (AFU) per nuclei (*n* = 3–4). (**C**) Cell viability, determined by trypan blue exclusion and expressed as % viable cells remained unchanged between CON vs. STIM treated myoblasts (*n* = 3). Data were analyzed using an unpaired Student’s *t* test, and are expressed as scatter plots with mean.

**Figure 7:**
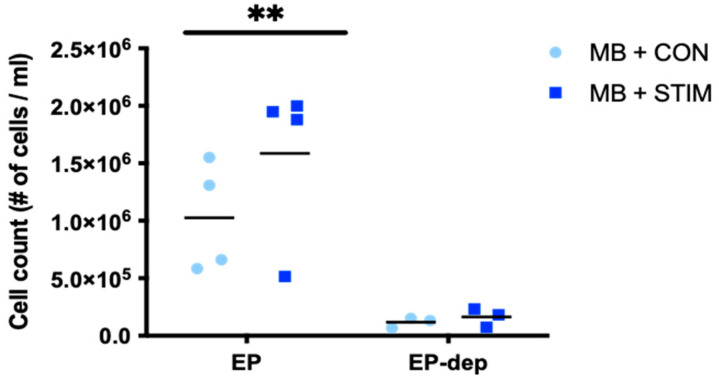
Cell count in myoblasts (MB) treated with EPs or EP-depleted media from CON vs. STIM myotubes. 90,000 cells/well were seeded in 6-well plates and left to adhere for 4 h. Cells were treated with 13 µg/well EPs (isolated using Total Exosome Isolation Reagent (ThermoFisher, Waltham, MA, USA) or with EP-depleted media (EP-dep, 1 mL) conditions from non-stimulated control (CON) vs. acutely stimulated (STIM) myotubes for 72 h. Total number of cells were counted using a haemocytometer and expressed per mL of media. Cell count was ~90% lower in myoblasts treated with EP-dep conditions from either CON or STIM myotubes (** *p* < 0.01, main effect of EP vs. EP-dep, *n* = 3–4).

It is well established in the literature that contractile activity induces an increase in mitochondrial biogenesis, and that skeletal muscle can release myokines/other factors to promote pro-metabolic adaptations in other tissues. Hence, our hypothesis was that EPs derived post-stimulation would play an important role in mediating this pro-metabolic effect originating from the contracting skeletal muscle. To mimic this crosstalk in vitro, we performed EP co-culture experiments to measure the potential of EPs after an acute stimulation to deliver an adaptive metabolic response in other cells. We seeded C2C12 myoblasts at 90,000 cells/well in a standard 6-well plate. Each well was incubated with 13 µg (6.67 µg/mL) of freshly isolated EPs from control or stimulated myotubes for 48 h and 72 h. This dosage was empirically determined as shown previously [42,60,61]. After treatment, we stained the cells for mitochondrial content with 0.1 mM MitoTracker CMXRos for 30 min. Representative images at 10X are shown for myoblasts treated with non-stimulated control and stimulated myotube-EPs for 48 h and 72 h (Figure 6A). No significant difference was found between the mitochondrial staining corrected by total nuclei count at either the 48 h or 72 h time point (Figure 6B). To determine the effect on cell viability, non-stimulated control or stimulated EP-treated myoblasts were counted using a trypan blue exclusion assay after 72 h treatment. Cell viability was not affected by non-stimulated control or stimulated myotube-EP treatment (Figure 6C). There was also no significant difference in total cell count at 72 h with stimulated myotube-EP vs. control treatment (Figure 7). However, myoblasts treated for 72 h with conditioned media that were depleted of EPs (EP-dep, 1 mL) displayed a ~90% decrease in cell count (Figure 7), irrespective of whether they were treated with non-stimulated control or stimulated EP-dep media.

### 3.4. Effect of Fetal Bovine Serum (FBS) and Horse Serum (HS) on Vesicle Preparations

C2C12 myoblasts were cultured in growth media (GM) containing 10% FBS, as previous research has shown that growing cells in exosome-depleted FBS negatively affects cell growth [62]. Similarly, myotubes were placed in differentiation media (DM) supplemented with 5% HS that was not EV-depleted. Given that both FBS and HS can contain EVs from source that can confound the data, we isolated EPs from GM and DM only, and compared them to the EPs isolated from myoblasts and myotubes, respectively. Media were placed in 6-well plates with no cells and incubated at 37 °C for 24 h to mimic the myoblast/myotube-conditioned media acquisition procedure. EP size, zeta potential and protein yield in particles isolated from GM- and DM-only conditions are shown in Table 1. We compared GM-EP biophysical characteristics to myoblast-EPs, and DM-EP biophysical characteristics to myotube-EPs using unpaired Student’s *t* tests. While EP size and zeta potential were not different, there was a 4.7-fold increase in protein yield in GM-EPs vs. myoblast-EPs (*p* < 0.05, Table 1). DM-EPs were 68% larger in average size, and had 72% less protein yield compared to myotube-EPs (*p* < 0.05, Table 1). A preliminary assessment of EV subtype marker protein expression (TSG101, CD81 and APO-A1), comparing myoblast-EPs and myotube-EPs to GM-EPs and DM-EPs, respectively, was performed (Supplementary Materials Figure S1). We noted two interesting observations: the pattern of expression from the cell (e.g., myotube-EPs) matched that of the EPs sourced from the culture media (e.g., DM-EPs). Second, the expression was much higher in cell-derived EPs than those obtained from just the media-EP preparations.

## 4. Discussion

Our results indicate that skeletal muscle myoblast differentiation into myotubes affects the vesicle size, protein yield and expression of subtype protein markers. Importantly statistically significant differences in vesicle biophysical characteristics (size and distribution) were observed with the crude method of particle isolation (TEI) but not with the gold standard method of EV isolation, dUC [19]. However, while dUC-derived EVs showed similar trends in differences between biophysical characteristics (size and distribution) of EVs from myoblasts vs. myotubes, these were not statistically significant. We expect the discrepancy is likely due to the smaller sample size in the dUC-derived EVs as well as the use of DLS, as discussed below. On the other hand, the difference in protein yield was observed in both TEI-derived particles and dUC-derived vesicles. Acute contractile activity had no effect on the biophysical properties of EPs and culturing myoblasts with control vs. stimulated myotube-EPs did not affect mitochondrial mass or cell viability/count. Interestingly, the addition of myotube-Eps, irrespective of whether the myotubes were stimulated or not, increased cell counts in treated myoblasts when compared to myoblasts treated with EP-depleted media only.

Previous work describing average size of myoblast and myotube EVs are largely consistent in their description of both cells releasing primarily small EVs [40,41,42,63,64]. Forterre et al. (2014) [41] reported myoblast- and myotube-EVs had similar average EV size, with no particles over 500 nm being found. However, in this study [41] the authors used a 0.2 µm filter before dUC so that would have removed any particles over 300 nm. Similarly, others using dUC and 0.2 µm filtration have reported average myoblast-EV size ranges between 50 and 100 nm [63,64]. The EVs in these studies were analyzed by either DLS [41], and/or nanoparticle tracking analysis (NTA) and transmission electron microscopy (TEM) analysis [63,64]. We observed myoblasts display a bimodal expression of EV sizes (<200 nm, and 400–600 nm), consistently in both TEI-derived EPs and dUC-derived EVs, without filtration in either method, and when biophysical properties were analyzed using DLS. Thus, it is likely that the discrepancy in sizes can be explained by the method of vesicle isolation and/or characterization. While DLS has been used in nanoparticle/EV research extensively, it is more effective for non-biological samples where the polydispersity index (PDI) is more or less constant and closer to 0.1 or lower. In biological samples such as myotube/myoblast-EV/EPs, PDI tends to be higher. In our current study, the EV/EP samples had intermediate polydispersity (<0.5), which is considered acceptable for size analysis, but still indicates that the EV/EP preparations were not homogenous and could have had aggregates. Finally, dUC is also known to cause EV aggregate formation due to the 100,000× *g* high speed spin step [19]. Together this could explain the absence of EVs below 150 nm and the high variability present in the data in Figure 2D. Further, in our study vesicle size was likely smaller in EPs vs. EVs, as TEI precipitates exomeres as well as other non-EV co-isolates that can affect the size profile, as detailed in MISEV guidelines [51]. While dUC is the preferred and no doubt the orthogonal approach for isolation of EVs compared to TEI-based precipitation, our results show similar trends in size and protein yield between the two methods, which is important for laboratories that do not have access to high speed ultracentrifugation infrastructure. Collectively, the data show that the EV isolation method in conjunction with the characterization methods are important factors to consider when analyzing EVs from skeletal muscle cells in culture.

We found that myotube-EPs expressed lower expression of small-EV markers (Alix, TSG10, CD63, CD81) and higher expression of microvesicle marker (ARF6) and non-EV co-isolate (APO-A1, lipoprotein marker) compared to myoblast-EVs. Romancino et al. (2013) [40] have postulated that myotube-EVs vs. myoblast-EVs may have different origins and that myotube-EVs are likely preferentially released through plasma membrane budding (microvesicles). This hypothesis supports the increased ARF6 expression we measured in myotube-EPs in our study. Previous proteomic analysis on myotube-EVs has indicated they contain less endosomal/lysosomal CD63, as opposed to CD81 or CD9 tetraspanins that are also expressed at the plasma membrane [41]. In line with this, our study showed myotube-EPs are CD63- and CD81+. However, we did not observe higher Alix or CD81 expression in myotube-EPs compared to myoblasts, as reported by others [40,41], nor similar levels of TSG101 expression [41], likely due to the differences in methodology noted above. As noted by others, expression of small EV/microvesicle markers proteins is cell-specific as well as dependent on isolation methodologies. Hence, expression alone cannot be used to precisely confirm vesicle origin or sub-type [51,65,66]. This highlights the heterogeneity of EPs isolated from skeletal muscle and again underscores the importance of careful documentation of the methods used to isolate and characterize vesicles.

Our results showed that myotube-EPs contained 15-fold more protein than myoblast-EPs when obtained using TEI. This difference between pre- and post-differentiated skeletal muscle cells remained in the dUC-derived EVs, although the magnitude was markedly reduced: myotube-EVs had 4.79-fold higher protein yield than myoblast-EVs. It is now well known that TEI allows for high recovery but low specificity of separated vesicles, whereas dUC permits intermediate to high specificity but low recovery of vesicles [51]. Consequently, we expected absolute protein yield to be higher in TEI-derived EPs, likely because TEI pulls down non-EV co-isolates and proteins. Given that myotube-EPs expressed significantly elevated levels of APO-A1, protein contamination from non-EV sources is likely amplifying protein yield in this method. Another source of contamination can be proteins from serum in the conditioned media preparations. This protein contamination from the media can be present in both myoblast-EP and myotube-EP isolates. Differentiating myotubes in serum-depleted media 24 h prior to vesicle isolation has been used to mitigate this problem [67]. However, growing myoblasts in exosome-depleted serum has been shown to negatively affect their proliferation and differentiation [68]. This indicates that an exosome- or serum-depleted approach may alter cell behavior and may not be advisable given the experimental context. In this study we chose not to use vesicle-depleted serum and instead established a baseline of the EP biophysical properties expected in media-only conditions. Encouragingly, we found that EPs isolated from growth or differentiation media only had significantly different protein yields compared to myoblast- and myotube-EP preparations, respectively. Differentiation media-derived EPs were also larger sized than myotube-EPs. The expression of proteins related to EV subtypes followed the same pattern for myoblast-EPs and myotube-EPs, as the EPs sourced from their respective culture media, as shown in Supplementary Figure S1. However, the expression was clearly higher in cell-derived EPs vs. media-derived EPs. This indicates that the confounding effect of media-derived proteins/particles on skeletal muscle–derived EP preparations in our study was likely limited but not completely abrogated. Thus, it is imperative to view results presented in Figure 1, Figure 2 and Figure 3 with the caveat that the EPs isolated are a mixture of both myoblast-/myotube-EPs as well as the respective serum-EPs in the media. However, for Figure 4, Figure 5, Figure 6 and Figure 7, where acute STIM induced myotube-EPs are evaluated against the non-stimulated controls, we believe the comparison is valid as both control and stimulated myotubes would theoretically have the same proportion of myotube-EPs and DM-EPs in the isolates, with the only difference being that of acute STIM. Moreover, many laboratories may lack access to highly specialized infrastructure such as high-speed ultracentrifuges or the financial capacity to purchase small EV–depleted serum from commercial sources. We believe our data provide relevant and important baseline information to consider in future assessment of EPs/EVs derived from skeletal muscle cells, pre- or post-differentiation into myotubes that are grown in standard media conditions. Lastly, the difference in protein yield between myotube-EVs vs. myoblast-EVs remained consistent when we used dUC to isolate the vesicles. Increased myotube-EV total protein content has also been reported by others previously [40]. This suggests that a significant increase in protein yield in myotube-EVs compared with myoblasts can be due to higher EV concentration, or enhanced EV protein cargo levels or a combination of both. We were not able to ascertain EV concentration due to lack of access to NTA or tunable resistive pulse sensing (TRPS)–based infrastructure for EV characterization at this point.

To our knowledge, this is the first characterization of EPs from myotubes after acute stimulation. Electrical pulse stimulation has been used extensively as a method of mimicking “exercise” and evoking the downstream effects associated with acute and chronic contractile activity in myotubes such as an increase in mitochondrial biogenesis [69]. However, it is important to remember the limitations associated with this model as discussed in the introduction. That said we compared our findings to previously reported data from exercise studies in rodents/humans and found alignment between our data and that of others as detailed next. That average EP size did not change after acute stimulation is not surprising. No change in average vesicle size has been reported after acute aerobic exercise in rats, or humans performing treadmill or cycle ergometer exercise [70,71,72]. Furthermore, the presence of small EVs after acute exercise in humans was reported to be 50–300 nm in size [46], in congruence with our results. However, each of the aforementioned studies reported an increase in small EV concentration after acute exercise. While we could not measure EV concentration in either the control or stimulation groups, our results show no significant differences in EV protein yield after acute stimulation. Furthermore, expression of small EV (TSG101, CD81) or microvesicle (ARF6) marker proteins did not change after acute stimulation. Previously, 1 h stimulation of C2C12 myotubes was shown to induce release of IL-6, a well-known myokine [39], as well as to increase cellular levels of proliferator activated receptor gamma coactivator 1 alpha (PGC-1α) and AMPK [73], indicating that 1 h of electrical stimulation is sufficient to induce myokine release in C2C12 myotubes, and evoke the cellular signaling milieu post-contractile activity. It is likely that a higher intensity or longer stimulation period would elicit changes in EV size or biophysical properties, but that needs to be experimentally determined. Additionally, acute exercise has been shown to increase CD61+ and CD81 + EVs [46] in human participants. Since our results showed no difference in CD81 expression, this may indicate that the source of increased CD81 expression is species-specific, or lies outside of skeletal muscle–derived EVs. Similarly, while acute exercise is known to increase circulating levels of microvesicles, these are predominantly platelet-derived [74], which supports our observations showing no difference in ARF6 expression between control and stimulated myotube-EPs. Further research with live animals/human subjects is warranted to determine the physiological context of our findings and identify the role of skeletal muscle–derived EVs. Despite all the limitations with this in vitro model of contractile activity, given the complexities associated with isolating skeletal muscle–derived EVs in vivo, and especially during exercise, this model offers some advantages. It provides us with the ability to measure the effects of contractile activity on skeletal muscle-EVs from skeletal muscle cells alone, and serves as a first step in the process of determining the importance of EVs during exercise.

Since EV cargo can be taken up by recipient cells, and can regulate their fate [16], we decided to evaluate the biological action of acute stimulation–derived EPs. To do so, we co-cultured myoblasts with control vs. stimulated myotube-EPs. After 48 h and 72 h EV treatment, we noted no significant effect of non-stimulated control vs. stimulated myotube-EPs on increasing mitochondrial mass as measured by MitoTracker staining. Increased mitochondrial content is a hallmark adaptation of contractile activity/exercise. Within hours of contractile activity, a cascade of reactions occur whereby PGC-1α is upregulated, which results in the downstream increase of nuclear respiratory factor 1 (NRF-1) and mitochondrial transcription factor A (Tfam), leading to a co-ordinated increase in both nuclear and mitochondrial proteins [55]. Within days to weeks of repeated contractile activity, mitochondria can populate the previously exercised muscle cell [73]. Since we measured mitochondrial content by mitochondrial staining, we did not assess if the aforementioned proteins associated with mitochondrial biogenesis could have been induced. This gap can be addressed in future studies. It is also likely that while treatment with stimulated myotube-EPs may trigger the protein signaling cascade upstream of mitochondrial biogenesis, a single dose is not powerful enough to elicit changes in organelle synthesis. EP treatment from chronically stimulated myotubes could help to fully elucidate the potential of skeletal muscle–derived vesicles to transmit and deliver an exercise response in non-stimulated cells. A growing number of studies have shown that skeletal muscle–derived EVs can have important paracrine effects that affect physiological function (e.g., myogenesis), as well as play a crucial rule in chronic diseases such as insulin-resistance, as reviewed comprehensively by others [74]. Our work is the first to characterize and subsequently perform co-culture with EPs from stimulated and non-stimulated control myotubes. This warrants further investigation into the biogenesis, uptake, and downstream biological activity of skeletal muscle–derived EVs, particularly with chronic exercise/contractile activity. Intriguingly, adding EPs from myotubes (irrespective of whether these myotubes were non-stimulated controls or acutely stimulated cells) increased cell count when compared to myoblasts that were treated with EP-depleted media. This indicates that myotube-EP preparations contain factor(s) that can potentiate cell growth and/or suppress cell death—two likely reasons behind the increased cell counts observed. Further research is required to fully understand the mechanisms underlying this observation and to ascertain whether this effect of myotube-EPs on cell count can be leveraged in a disease-related cellular context.

We acknowledge the additional limitations in our current study that were not already noted above. Polyethylene glycol (PEG)–based TEI kit-based precipitation of EPs is known to be a high yield, low purity method of vesicle isolation [51]. Proteins from the media may be pulled into the isolated EP pellet and may mask/confound the true difference in EP preparations. To further address this, future work analyzing the proteomic content of purified myoblast- and myotube-EVs and their potential biological activity is warranted. Additionally, characterizing STIM and non-stimulated control EVs for proteomic, genomic and lipidomic content would give a better understanding of vesicle differences immediately after acute stimulation. We only measured mitochondrial staining as a proxy for mitochondrial mass/content. Evaluating changes in signaling cascades and protein expression/translocation that are upstream of an increase in mitochondrial content could provide deeper insight into whether stimulation derived myotube-EPs can evoke a metabolic response in treated cells. Lastly, experiments with longer treatment time, higher dosage and duration of contractile activity, i.e., with EPs derived post-chronic contractile activity, can provide a better understanding of the role of skeletal muscle–derived EVs in conferring any of the adaptive metabolic changes commonly associated with contractile activity.

## Figures and Tables

**Table 1:** Average size, zeta potential and protein yield of EPs isolated from myoblasts (MB) and myotubes (MT) and their respective media-only conditions. C2C12 myoblasts were cultured in growth media (GM) made from DMEM containing 10% fetal bovine serum (FBS) and 1% antibiotics. To differentiate myoblasts into myotubes, media was switched to differentiation media (DM): DMEM supplemented with 5% horse serum (HS) and 1% antibiotics. FBS and HS can contain EVs from bovine or horse serum, respectively. To evaluate the confounding effect of the serum (if any), EPs were isolated using Total Exosome Isolation Reagent (ThermoFisher, Waltham, MA, USA) from GM-only conditions and compared to myoblast-EPs, and from DM only and compared to myotube-EPs (*n* = 3–6, * *p* < 0.05). Results were analyzed using an unpaired Student’s *t* test. Data are expressed as mean ± standard error.

Sample	Average Size	Zeta Potential (mV)	Protein Yield (μg)
Myoblast-Eps	167.30 ± 12.21	−11.3 ± 2.74	31.61 ± 1.84
GM-Eps	218.2 ± 52.68	−18.31 ± 4.76	148.2 ± 73.73
*t*-test (GM-Eps vs. Myoblast-Eps)	NS, *p* = 0.167	NS, *p* = 0.243	* *p* = 0.020
Myoblast-Eps	98.5 ± 7.86	−16.21 ± 3.55	472.6 ± 73.71
DM-Eps	165.9 ± 28.64	−11.68 ± 2.29	129.70 ± 13.99
*t*-test (DM-Eps vs. Myoblast-Eps)	* *p* = 0.024	NS, *p* = 0.360	* *p* = 0.003

## Data Availability

Not applicable.

## Supplementary Materials

The following supporting information can be downloaded at: https://www.mdpi.com/article/10.3390/membranes12050464/s1, Figure S1: Expression of EV subtype protein markers in EPs from differentiation media (DM), growth media (GM) compared to myotube-EPs and myoblast-EPs, respectively.
