# Supplementary material for: Characterizing Extracellular Vesicles and Particles Derived from Skeletal Muscle Myoblasts and Myotubes and the Effect of Acute Contractile Activity
Source: Membranes (Basel). 2022 Apr 26;12(5):464. doi: 10.3390/membranes12050464 (PMC9144336; doi:10.3390/membranes12050464)
Supplement: Supplementary file 1 [file membranes-12-00464-s001.zip › membranes-1629922-supplementary.pdf]

Supplementary material

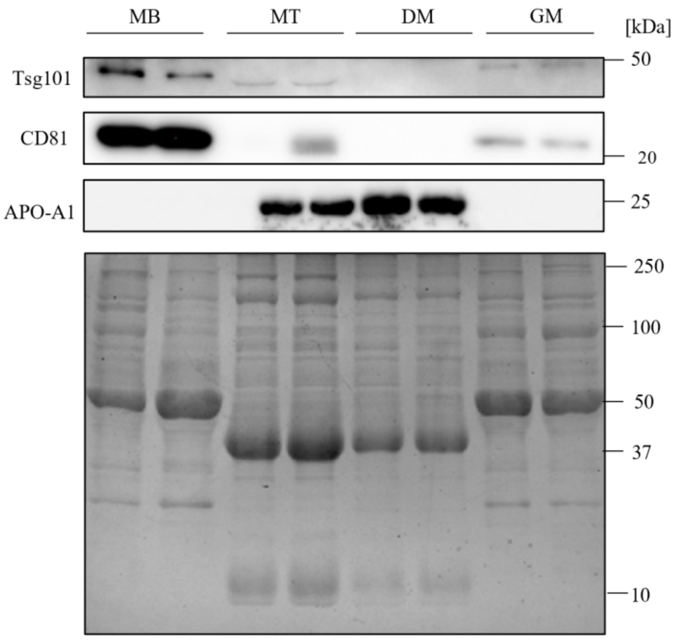

Figure S1. Expression of EV subtype protein markers in EPs from differentiation media (DM), growth media (GM) compared to myotube-EPs and myoblast-EPs, respectively.

Representative immunoblots showing equal amounts of myoblast (MB)-EPs, myotube (MT)-EPs, DM-EPs and GM-EPs. EP lysates were subjected to 12% SDS-PAGE, and probed with antibodies against TSG101, CD81 and APO-A1. Coomassie Blue gel staining was used as loading control (N=2/condition). We noted two interesting observations: (i) the Coomassie stain, and TSG101, CD81 and APO-A1 from the media-derived EPs follow the pattern observed in the respective cell types e.g. myoblast-EPs have higher expression of Tsg101 and CD81 than myotube-EPs and GM-EPs have a higher expression of Tsg101 and CD81 than DM-EPs. Similarly, APO-A1 is elevated in both myotube-EPs and DM-EPs, compared to myoblast-EPs and GM-EPs, respectively. (ii) protein expression was overall much higher in cell-derived EPs than the ones sources from culture media. There is a clear overlap in the EPs derived from the media only conditions and the EPs from the cells which indicates some degree of contamination from serum-derived EPs in our preparations. Future investigations should carefully analyze the proportion of EPs from media, especially if EV-depleted serum formulations are not used.
